# Reply to: Perfusion index for assessing microvascular reactivity in septic shock after fluid resuscitation

**DOI:** 10.5935/0103-507X.20190042

**Published:** 2019

**Authors:** Igor Alexandre Côrtes Menezes, Cláudio Leinig Pereira da Cunha, Hipólito Carraro Júnior, Alain Marcio Luy

**Affiliations:** 1 Departamento de Clínica Médica, Hospital de Clínicas, Universidade Federal do Paraná - Curitiba (PR), Brasil.; 2 Unidade de Terapia Intensiva, Hospital de Clínicas, Universidade Federal do Paraná - Curitiba (PR), Brasil.

**To the Editor**

We thank Drs Daş, Bardakc and Beyazıt for their insightful comments on our recent article in " *Revista Brasileira de Terapia Intensiva* " regarding the clinical use of the perfusion index (PI) to assess microvascular reactivity in septic shock.^(1)^ As they highlighted in their letter, some factors can potentially influence the evaluation of perfusion index. As previously cited the PI is very sensitive to sympathetic stimuli.^(2)^ This was confirmed by our results, which showed a positive correlation of PI values after vascular reactivity tests with noradrenaline doses. The ambient temperature is another important factor, since cutaneous tissue, whose microcirculation is evaluated by PI, is also important in thermoregulation and influenced by sympathetic stimuli.^(3)^ As reported in the study, all patients were evaluated under controlled temperature (25ºC) in order to reduce the influence of this factor.

Regarding the position of the patients, although only recently it has been verified as influential in PI, there was previous knowledge about the sympathetic stimuli in the auto-regulation of the positional blood flow.^(4)^ So we also take certain precautions when evaluating this factor. All patients with septic shock were maintained in a supine position between 30 and 45 degrees of head elevation (data not mentioned in the article). In addition, PI assessment was performed after resuscitation and at least 1 hour hemodynamic stability. During this period and throughout the evaluation, the patients were not moved. In relation to the control group, the same care was taken. Because of this care, we believe that the patient's position did not substantially influence the interpretation of our results. However, we appreciate this comments on this factor and others that point out that the assessment of peripheral perfusion with the PI is not as simple as it seems at first glance, despite the ease of use of the method.

*Igor Alexandre Côrtes Menezes Department of Internal Medicine, Hospital de Clínicas, Universidade Federal do Paraná -Curitiba (PR), Brazil.* *Cláudio Leinig Pereira da Cunha Department of Internal Medicine, Hospital de Clínicas, Universidade Federal do Paraná -Curitiba (PR), Brazil.* *Hipólito Carraro Júnior Intensive Care Unit, Hospital de Clínicas, Universidade Federal do Paraná -Curitiba (PR), Brazil.* *Alain Marcio Luy Intensive Care Unit, Hospital de Clínicas, Universidade Federal do Paraná -Curitiba (PR), Brazil.*
